# The exploration of new biomarkers for oral cancer through the ceRNA network and immune microenvironment analysis

**DOI:** 10.1097/MD.0000000000032249

**Published:** 2022-12-09

**Authors:** Sai Ma, Jie Guo, Xuan Zhang, Yongchao Yang, Yang Bao, Suxin Zhang, Tianke Li

**Affiliations:** a The First Affiliated Hospital of Hebei North University, oral and maxillofacial surgery, Zhangjiakou, Hebei Province, China; b The Fourth Hospital of Hebei Medical University, Department of Stomatology, Shijiazhuang, Hebei Province, China.

**Keywords:** biomarkers, ceRNA network, immune microenvironment, oral cancer

## Abstract

The competitive endogenous RNA (ceRNA) and tumor-penetrating immune cells may be related to the prognosis of oral cancer. However, few studies have focused on the correlation between ceRNAs and immune cells. Thus, we developed a method based on a ceRNA network and tumor-infiltrating immune cells to elucidate the molecular pathways that may predict prognosis in patients with oral cancer. Download RNAseq expression data of oral cancer and control samples from the Cancer Genome Atlas (TCGA), obtain differentially expressed genes and establish a ceRNA network. The cox analysis and lasso regression analysis were used to screen key RNAs to establish a prognostic risk assessment model, and draw a 1.3.5-year forecast nomogram. Then the CIBERSORT algorithm was used to screen important tumor immune infiltrating cells associated with oral cancer. Another prognostic predictive model related to immune cells was established. Finally, co-expression analysis was applied to explore the relationship between key genes in the ceRNA network and important immune cells. Multiple external data sets are used to test the expression of key biomarkers. We constructed prognostic risk models of ceRNA and immune cells, which included 9 differentially expressed mRNAs and 2 types of immune cells. It was discovered from the co-expression analysis that a pair of important biomarkers were associated with the prognosis of oral cancer. T cells regulatory and CGNL1 (*R* = 0.39, *P* < .001) showed a significant positive correlation. External data set validation also supports this result. In this study, we found that some crucial ceRNAs (GGCT, TRPS1, CGNL1, HENMT1, LCE3A, S100A8, ZNF347, TMEM144, TMEM192) and immune cells (T cells regulatory and Eosinophils) may be related to the prognosis of oral cancer.

## 1. Introduction

Oral cancer is 1 of the 10 most prevalent cancers in the world, with a 5-year survival rate of approximately 50%. Oral cancer includes malignant tumors of the cheeks, tongue, gums, floor of the mouth, and hard palate. About 95% of oral malignancies are squamous cell carcinoma.^[1]^ Smoking, alcohol consumption, and viral infections are all causes of oral squamous cell carcinoma, among which smoking and drinking are the 2 main causes of the high incidence of oral squamous cell carcinoma.^[2]^ Human papillomavirus is a high-risk factor for oral squamous cell carcinoma, and HPV16 has been listed as one of the pathogenic factors of oral squamous cell carcinoma by the international agency for research on cancer.^[3]^ In addition, HPV33, HPV35 and others (HPV viruses in cervical cancer) are also considered to induce oral squamous cell carcinoma.^[4]^ Oral cancer is characterized by late diagnosis, poor prognosis, low overall survival (OS) rate and easy local recurrence.^[5]^ The reason for the high mortality from oral cancer can be summed up as the difficulty in early diagnosis or the lack of specific indicators, which makes it difficult to predict the development of the tumor and the prognosis of the patient.^[6]^ Researchers and doctors are constantly exploring new biomarkers to diagnose cancer as early as possible, thus extending the survival of oral cancer patients.^[7]^

In recent years, there is growing evidence that the pattern of mutual regulation mode between lncRNA and miRNA and its downstream target genes is closely related to the occurrence and development of tumors. As an essential transcriptional regulator, the activity of miRNA can be regulated by adsorption of lncRNA. This type of RNA is also referred as competitive endogenous RNA (ceRNA).^[7]^ LncRNA is competitively combined with miRNA to affect protein levels in encoded genes and to participate in the regulation of cellular biological behavior. Previous studies have revealed ceRNAs involved in the growth, metastasis and prognosis of oral cancer, such as HOXA10-AS, FGD5-AS1, etc.^[8–10]^ In this study, different expressions of RNAs (including lncRNAs and mRNAs) were screened to construct ceRNA networks and probe potential markers related to disease prognosis.

Tumor osmosis of immune cells is believed to play a key role in the growth, progression and metastasis of cancer cells.^[11]^ Human papillomavirus is a high risk factor for oral squamous cell carcinoma. The immune system attach importance to the control of viral infections, which provides the feasibility for immunotherapy of oral cancer.^[12]^ Although the initial results of immunotherapy have been achieved, the overall response rate is not satisfactory, and there are shortcomings of high treatment cost and potential immune-related deficiencies. The research on the immune microenvironment of oral cancer is particularly vital.^[13]^ There have been studies on the immune microenvironment of oral cancer.^[12]^ However, few studies have focused on the regulatory mechanism between the ceRNA network and tumor-permeable immune cells.

**Figure 1.**
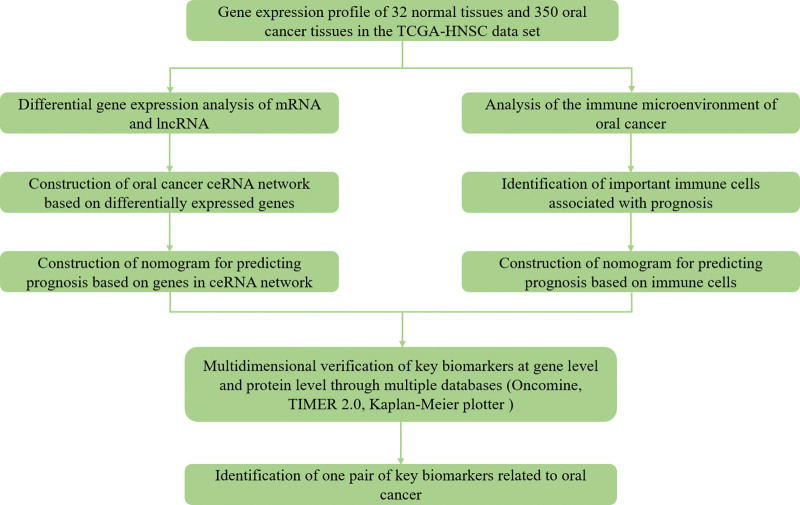
The flow chart of the whole research process.

In this study, we constructed a ceRNA network to examine the underlying molecular mechanisms of oral cancer. The prognosis of oral cancer may be related to both the ceRNA network and the type of tumor infiltrating immune cells, thus we applied the Cibersort algorithm to assess the proportion of immune cell types in tumor samples and quantify the cellular composition of the immune responses. On the basis of the prognostic models of the ceRNA network and immune cells, a nomogram is constructed to predict the prognosis of oral cancer. In addition, we evaluated the relationship between tumor-infiltrating immune cells and ceRNA networks, which provided insight into the molecular mechanisms and clinical predictors of oral cancer. A flow chart explaining this process is provided in Figure 1.

## 2. Materials and Methods

### 2.1. Data set acquisition and differential expression analysis

This study was endorsed by the Ethics Committee of the Fourth Hospital of Hebei Medical University. Oral cancer RNA-seq raw count and fragments per kilobase per million mapped reads and miRNA-seq data were downloaded from the Cancer Genome Atlas (TCGA) database (https://cancergenome.nih.gov/).^[14]^ The experimental group and the control group in this study were 350 cases of cancer tissues and 32 cases of normal tissues adjacent to cancer. We also retrieved the demographic information (age, gender), survival endpoint (vital status, days to death) and stage, TNM, and grade of each patient (Table 1).^[15]^ Differentially expressed genes between the experimental group and the control group was analyzed using edgeR package in the R software. Up or downregulated genes were defined as false discovery rate adjusted *P* < .01 and log fold change > 2.0 or < −2.0. edgeR was specially designed to analyze raw RNA-seq expression data to calculate differential gene expression between cancer tissues and normal tissues adjacent to cancer.^[16]^

**Table 1:** Clinical information statistics of TCGA dataset.

Characteristic		TCGA dataset (n = 382)
Survival status	Alive	203
	Dead	162
Age	<=30	8
	30~60	152
	>=60	204
Sex	Female	114
	Male	251
Grade	G1	54
	G2	221
	G3	76
	G4	4
T	T1	35
	T2	107
	T3	73
	T4	119
N	N0	131
	N1	53
	N2	117
	N3	4
M	M0	135
	MX	44
Stage	I	22
	II	58
	III	65
	IV	182

TCGA = the cancer genome atlas.

### 2.2. *Construction of* ceRNA *network*

We use mircode database to identify the interaction between lncRNA and miRNA (http://www.mircode.org/).^[17]^ The miRNA-mRNA interaction is obtained from miRTarBase database (https://mirtarbase.cuhk.edu.cn/).^[18–20]^ We searched for miRNA with DElncRNA (differentially expressed lncRNA) as the target in mircode database, and for target genes of these miRNAs in miRTarBase, which were intersected with DEmRNA (differentially expressed mRNA). Then, the Pearson correlation coefficient was calculated and the correlation between lncRNA and mRNA was screened. We used Cytoscape v.3.7.2 software to select the miRNAs that regulate lncRNAs and mRNAs, which were significant in the hypergeometric test and correlation analysis. These were used for visualizing the ceRNA network. mRNAs in the ceRNA network are the parts that perform biological functions directly. Gene ontology and Kyoto Encyclopedia of Genes and Genomes enrichment analysis of DEmRNAs were carried out using the Metascape database (https://metascape.org/). The top 20 terms and pathways were selected according to the false discovery rate value from small to large.

### 2.3. *Construction of the prognostic risk model of key genes in the* ceRNA *network*

Univariate cox risk regression analyses were performed for the genes in the ceRNA network to identify key genes related to prognosis. We applied survival coxph function in R software to implement, selecting log rank *P* < .05 as the threshold. After that, 350 oral cancer samples were randomly divided into 2 groups. Overall, 75% of the samples were used as the training set and 25% as the test set. A lasso regression model was constructed from the training set, and the best lambda value and gene set were obtained. We use the glmnet package in R software to perform lasso cox regression analysis. The change trajectory for each independent variable is shown in Supplemental Digital Content (Fig. S1, http://links.lww.com/MD/I96). It can be seen that with the gradual increase of lambda, the independent variable coefficient tends to increase gradually. We use 10-fold cross-validation to test the accuracy of the model and obtain the confidence interval under each lambda. The model is optimal when lambda = 0.0268. At this point, we selected 18 genes for multivariate cox regression analysis. The 9 mRNAs with the smallest Akaike information criterion (AIC) value (AIC = 1565.73) was retained as the final model. Then, the lifetime of the test set was predicted by the constructed model, and receiver operating characteristic (ROC) curve were drawn. The cox risk regression model was constructed with these genes. We calculated the risk score of each sample according to the level of expression of the sample. Patients were divided into high-risk groups and low-risk groups based on the median risk score. The Kaplan–Meier method was used to analyze differences in the OS between the high-risk group and the low-risk populations. In addition, the timeROC package of R software was used to plot ROC curves of 1-, 3-, and 5-years. The rms package was used to draw nomograms.

### 2.4. Immune microenvironment analysis

Cibersort algorithm is a bio-informational method that uses RNA-seq count data to estimate the abundance of 22 different types of immune cells (http://cibersort.stanford.edu/).^[21]^ We used Cibersort package in R software to calculate the abundance of 22 immune cells in adjacent tissues and oral cancer tissues. *P* < .05 indicates that the difference is statistically significant. In addition, we use corrplot package and vioplot package in R to visualize the results.

Univariate cox risk regression analyses were performed for 22 immune cells to identify key immune cells related to prognosis. We use the survival coxph function in R software to implement, and select log rank *P* < .05 as the threshold. After that, 350 oral cancer samples were randomly divided into 2 groups. Overall, 75% of the samples were used as the training set and 25% as the test set. A lasso regression model was constructed from the training set, and the best lambda value and immune cell set were obtained. We use the glmnet package in R software to perform lasso cox regression analysis (Supplemental Digital Content [Fig. S2, http://links.lww.com/MD/I97]). Finally, the prognostic risk model of immune cell set was constructed by using multivariable cox analysis. The risk score for each sample can be calculated, and patients are divided into high-risk groups and low-risk groups based on the median risk score. The Kaplan–Meier method was used to analyze the differences in OS between the high-risk group and the low-risk group. The timeROC package of R software was used to draw ROC curves of 1, 3, and 5 years. The rms package was used to plot nomograms. In addition, we use the ggpubr package in R to explore the relationship between immune cells and clinical characteristics (age, grade, TNM).

We used the corrplot package in R to analyze the correlation between key genes in the ceRNA prognostic models and key immune cells in the immune cell prognosis model. Based on Pearson correlation analysis, a co-expressed heatmap was drawn to show the correlation of various immune cells and genes. The ggplot package in R is used to plot correlation curves of highly relevant genes and immune cells.

### 2.5. Multidimensional validation

To minimize bias, multiple databases which included Oncomine, TIMER2.0 database and Kaplan–Meier plotter were utilized to uncover gene expression levels of key biomarkers. The oncomine database integrates RNA-seq data from the Gene Expression Omnibus database, TCGA database, and published literature to analyze the expression level of genes in multiple types of cancers (https://www.oncomine.org/resource/login.html#).^[22]^ We used the TIMER2.0 database to analyze the differentiated expressions of key genes in different histological types of cancer and normal tissues. Kaplan–Meier plotter (http://kmplot.com/analysis/index.php?p=service) is an online survival analysis. Currently, the site can conduct research on 54,675 genes and 18,674 cancer samples, involving breast cancer, lung cancer and so on. On this basis, we analyzed whether the survival time of patients with different expression levels of key genes was significantly different.

## 3. Results

### 3.1. Screening of differentially expressed genes and construction of cerna network

**Figure 2.**
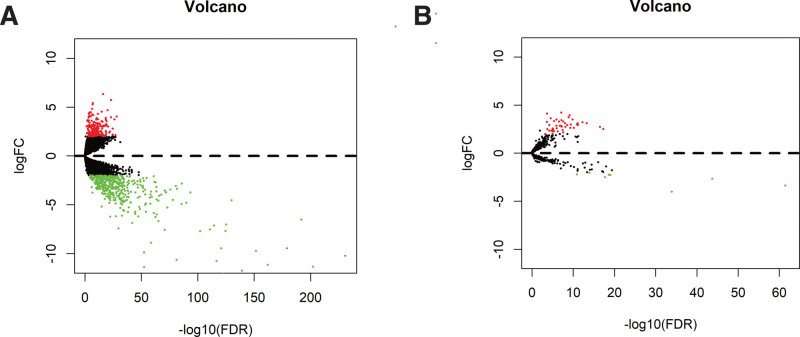
(A) The volcano map of differentially expressed mRNAs between normal and oral cancer tissues. (B) The volcano map of differentially expressed lncRNAs between normal and oral cancer tissues.

**Figure 3.**
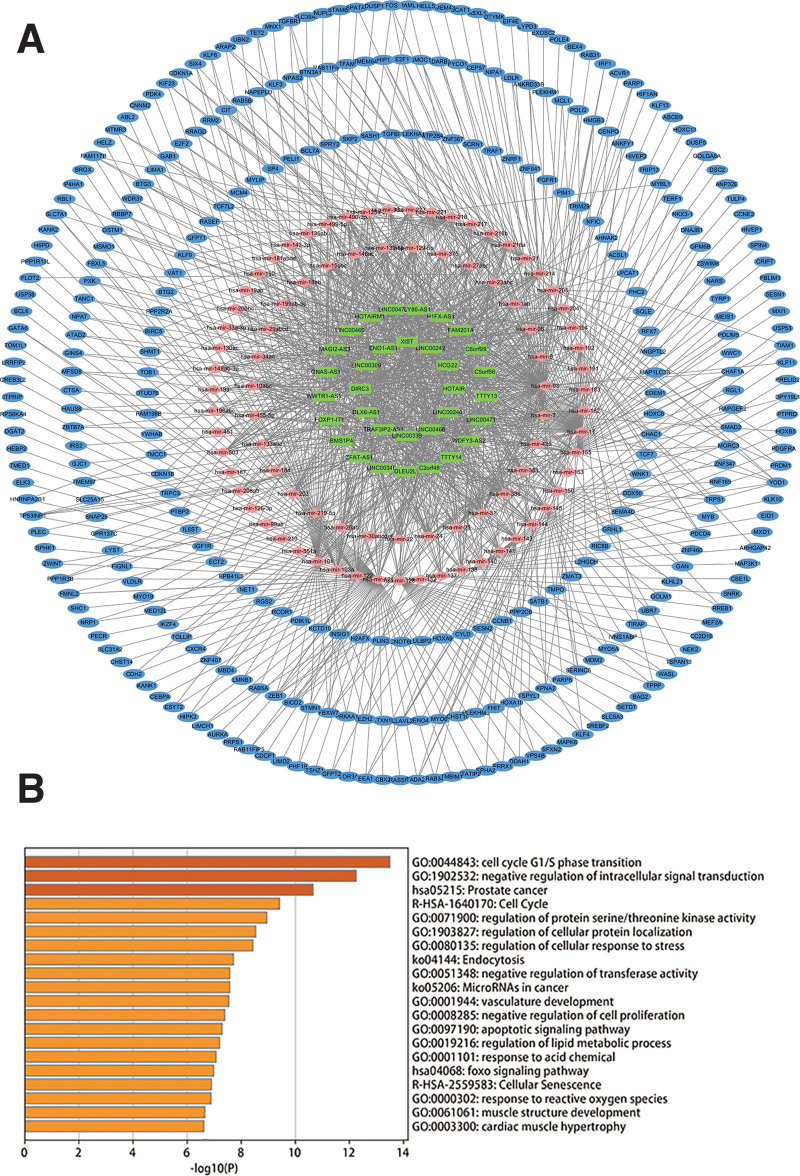
(A)The ceRNA network constructed by differentially expressed mRNA, lncRNA. Blue node refers to mRNA, green node refers to lncRNA, red node refers to miRNA. The gray line represents the interaction between them. (B) Enrichment analysis results of ceRNA network. ceRNA = competitive endogenous RNA.

We obtained differentially expressed genes from 32 normal tissues and 350 oral cancer tissues in the TCGA-HNSC dataset. As shown in Figure 2A and Supplemental Digital Content (Fig. S3A, http://links.lww.com/MD/I98), compared with normal samples, oral cancer samples have a total of 599 DEmRNAs (200 up-regulated, 399 down-regulated) and 53 DElncRNAs (45 up-regulated, 8 down-regulated) (Fig. 2B and Supplemental Digital Content [Fig. S3B, http://links.lww.com/MD/I98]). The shared miRNAs of differentially expressed mRNA and lncRNA were obtained from the mircode and miRTarBase databases, the Pearson’s correlation coefficient of lncRNA-mRNA pairs with shared miRNA was calculated, and the positive expressed lncRNA-mRNA pairs were screened. With Cytoscape 3.7.2 software, we constructed a ceRNA network of 454 nodes and 1319 edges, including 33 lncRNAs, 86 miRNAs and 335 mRNAs (Fig. 3A). Gene ontology and Kyoto Encyclopedia of Genes and Genomes enrichment analysis presented that the biological function of the ceRNA network was mainly concentrated in the cell cycle G1/S phase transition, negative regulation of intracellular signal transduction, prostate cancer, cell cycle, regulation of protein serine/threonine kinase activity, regulation of cellular protein localization, regulation of cellular stress response, endocytosis, negative regulation of transferase activity, microRNA in tumors (Fig. 3B).

### 3.2. *Construction and analysis of the prognostic risk model of key genes in the* ceRNA *network*

**Figure 4.**
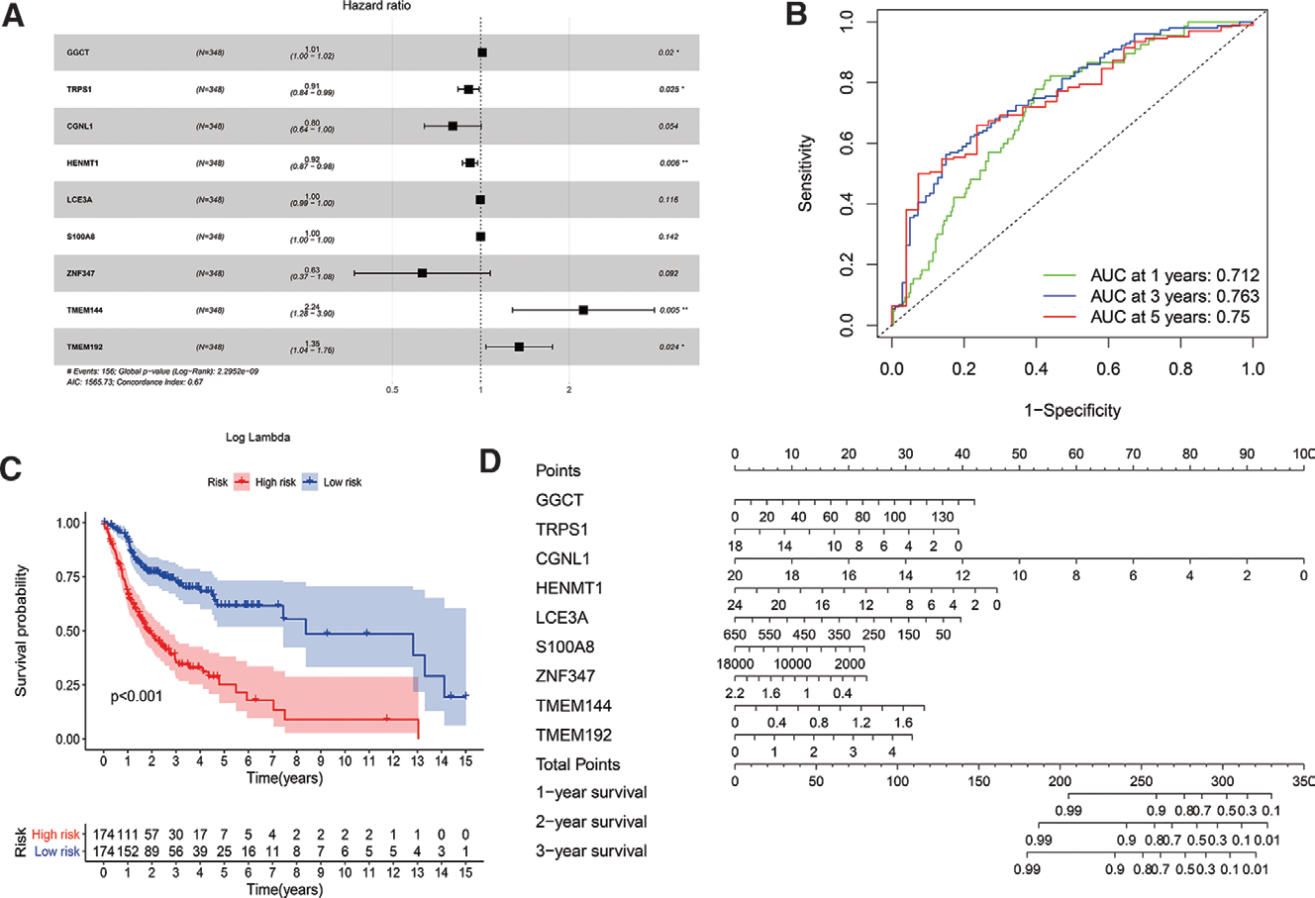
(A) The results of the multivariate cox regression (B) 1-, 3-, 5-year ROC curves of the constructed prognostic model. (C) The Kaplan–Meier survival curve of high-risk and low-risk patients. (D) The nomogram for predicting 1-, 2-, 3-year survival rates of oral cancer patients. ROC = receiver operating characteristic curve.


First, we performed univariate cox regression analysis of the expression of DElncRNAs and DEmRNAs in the ceRNA networks and the survival time of the samples. The results showed that there were 113 DEGs affecting the prognosis. We use the glmnet package in R software to perform lasso cox regression analysis. Finally, we selected 9 mRNAs with the smallest AIC value (AIC = 1565.73) for multivariate Cox regression analysis (Fig. 4A). The final 9-mRNA signature formula is as follows:

$$\begin{array}{l} \begin{array}{ll} RiskScore=\;\;\;\; & 0.0122\times exp^{GGCT}-0.0949\times exp^{TRPS1}\\ & -\;0.219\times exp^{CGNL1}-0.0836\times exp^{HENMT1}\\ & -\;0.00265\times exp^{LCE3A}-0.000549\times exp^{S100A8}\\ & -\;0.458\times exp^{ZNF347}+0.805\times exp^{TMEM144}\\ & +\;0.302\times exp^{TMEM192} \end{array} \end{array}$$

In accordance with the median risk score, patients were divided into high-risk groups and low-risk groups. Furthermore, we analyzed the prognostic classification efficiency of the model for 1-, 3-, and 5-years from RiskScore. As shown in Figure 4B, it can be seen that the model has a large area under the AUC curve. The AUC values for 1-, 3-, and 5-years are all above 0.7. Moreover, the Kaplan–Meier method was used to draw a survival curve for the 2 groups of patient samples, it was found that the OS rate of the high-risk group and the low-risk group was significantly different (*P* < .001). The survival rate of patients in the high-risk group was significantly lower than that in the low-risk group (Fig. 4C). A nomogram is established based on the results of the multivariate cox analysis, as shown in Figure 4D. These results indicate that RiskScore can effectively screen patients with high-risk oral cancer with poor clinical prognostics.

### 3.3. Analysis of the immune microenvironment of oral cancer

**Figure 5.**
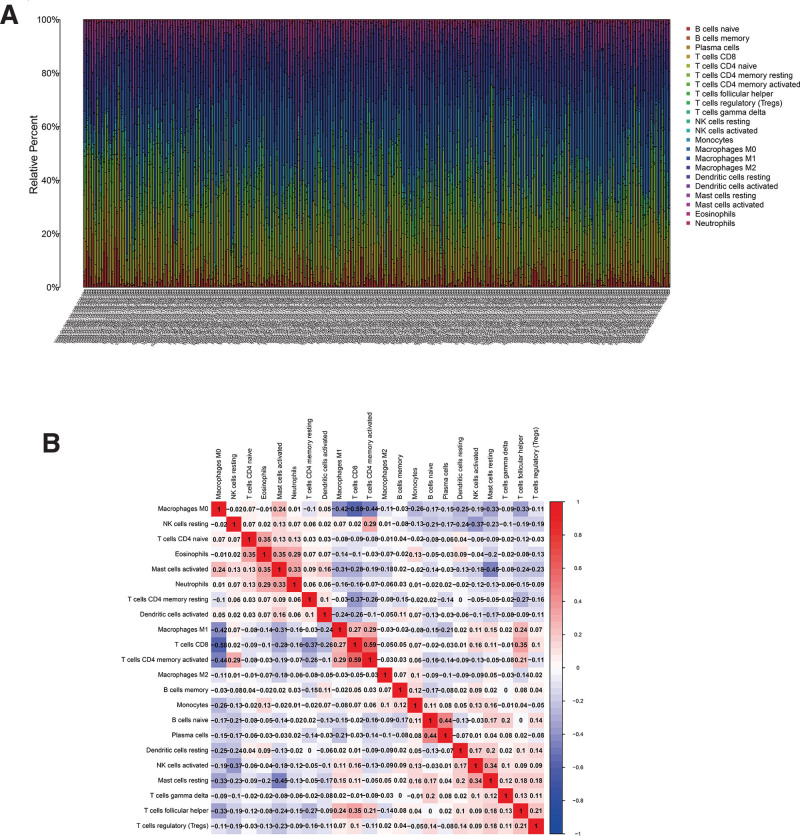
(A) The composition of immune cells in oral cancer evaluated by the CIBERSORT algorithm. (B) The result of the correlation analysis between significant immune cells.

**Figure 6.**
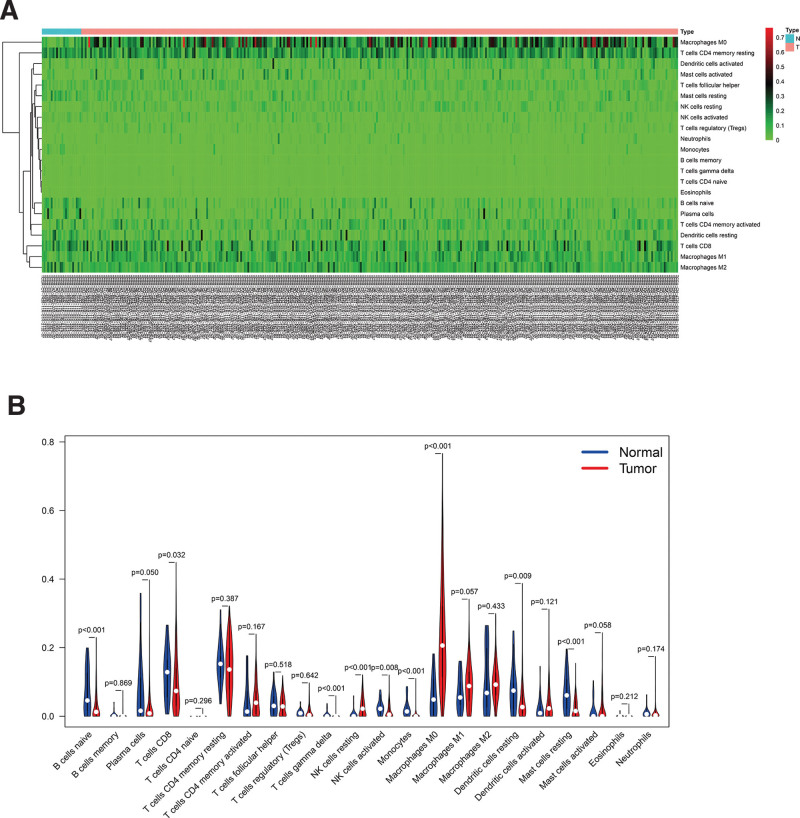
(A) Heat map of the distribution of immune cells in oral cancer and normal samples. (B) Distribution differences of immune cells between oral cancer and normal samples.

In order to further study the tumor immune micro-environment, we used the Cibersort package in R software to evaluate the immune infiltration between normal and tumor tissues. The relative percentages of 22 immune cells are shown in Figure 5A. Correlation analysis between immune cells showed a positive correlation between T cell CD4 naivety and eosinophils; Eosinophils, Neutrophils, and Mast cells activated were positively correlated; T cells CD4 memory activation, T cell follicle helper, and T cell CD8 were positively correlated. Macrophages M1, T cells CD8, T cells CD4 memory activated, Mast cells resting, and T cells follicular helper are negatively correlated with Macrophages M0; NK cells activated and NK cells resting are negatively correlated; Mast cells resting, Macrophages M1 and Mast cells activated There was a negative correlation; T cells CD8 and T cells CD4 memory resting showed a negative correlation (Fig. 5B). As shown in the heatmap and violin chart, B cells naive, T cells CD8, T cells gamma delta, NK cells resting, NK cells activated, Monocytes, Macrophages M0, Dendritic cells resting and Mast cells resting are significant difference in normal and tumor tissues (Fig. 6).

Survival analysis showed that B-cell naive, T cells regulatory and Eosinophils were significantly related to the prognosis of oral cancer (Supplemental Digital Content [Fig. S4, http://links.lww.com/MD/I99]). Samples with a low proportion of eosinophils had better survival status (logrank test, *P* = .007); samples with a high proportion of B-cell naive, T cells regulatory had better survival status (log-rank test, *P* = .041, *P* = .032).

The relationship between clinical features and immune cells is shown in Supplemental Digital Content (Fig. S5, http://links.lww.com/MD/I100). The results showed that Macrophages M0 was significantly overrepresented in stage IV samples compared with stage I samples (*P* = .024). Similarly, T cells regulatory was significantly overrepresented in stage I samples compared with stage IV samples (*P* = .003). T cells follicular helper was significantly overrepresented in N0 samples compared with N2 samples (*P* = .024). Plasma cells were significantly overrepresented in G1 samples compared with G3 samples (*P* = .008).

### 3.4. Construction and analysis of prognostic risk models related to immune cells

**Figure 7.**
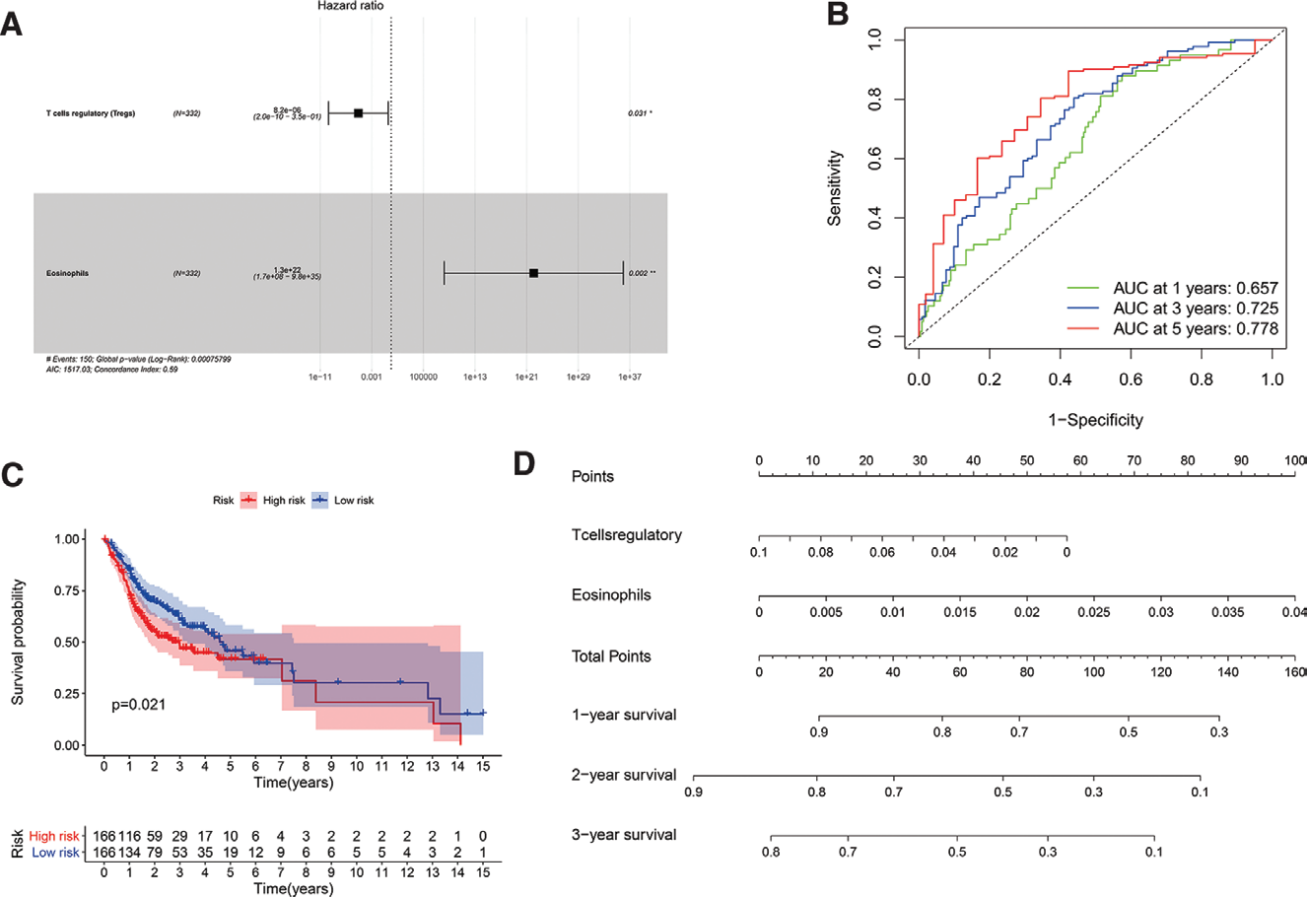
(A) The results of the multivariate cox regression (B) 1, 3, 5-year ROC curves of the constructed prognostic model. (C) The Kaplan–Meier survival curve of high-risk and low-risk patients. (D) The nomogram for predicting 1, 2, 3-year survival rates of oral cancer patients. ROC = receiver operating characteristic curve.

We performed multivariate cox regression analysis for the 22 different types of immune cells, and showed that the model composed of T cells regulatory and Eosinophils had the smallest AIC (AIC = 1517.03) (Fig. 7A). Two types of immune cells were selected to construct the multiple cox risk regression model and calculate the risk ratio. The ROC curve shows the accuracy of the model (1-year AUC: 0.657; 3-year AUC: 0.725; 5-year AUC: 0.778) (Fig. 7B). The Kaplan–Meier method was used to draw a survival curve for the 2 groups of patient samples, it was found that the OS rate of the high-risk group and the low-risk group was significantly different (*P* = .021). The survival rate of patients in the high-risk group was significantly lower than that in the low-risk group (Fig. 7C). The nomogram shows the effect of 3 key immune cells on the survival rate of patients at 1-, 2-, and 3-years (Fig. 7D). These results indicate that RiskScore can effectively screen patients with high-risk oral cancer with poor clinical prognostics.

### 3.5. Co-expression analysis of immune cells and key genes

**Figure 8.**
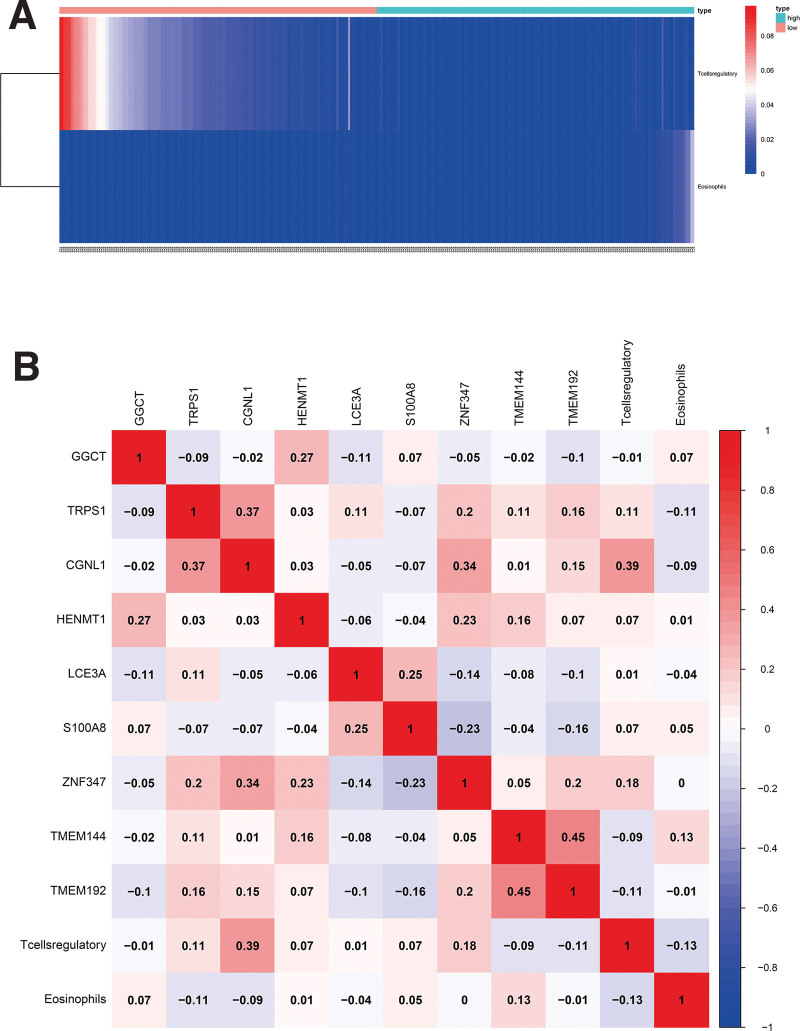
(A) The distribution of important immune cells in the high-risk group and the low-risk group. (B)The result of the co-expression analysis between tumor-infiltrating immune cells and key members of ceRNA network. The co-expression heatmap illustrated some significant co-expression patterns about key members in the ceRNA network and key members in the immune cells. ceRNA = competitive endogenous RNA.

**Figure 9.**
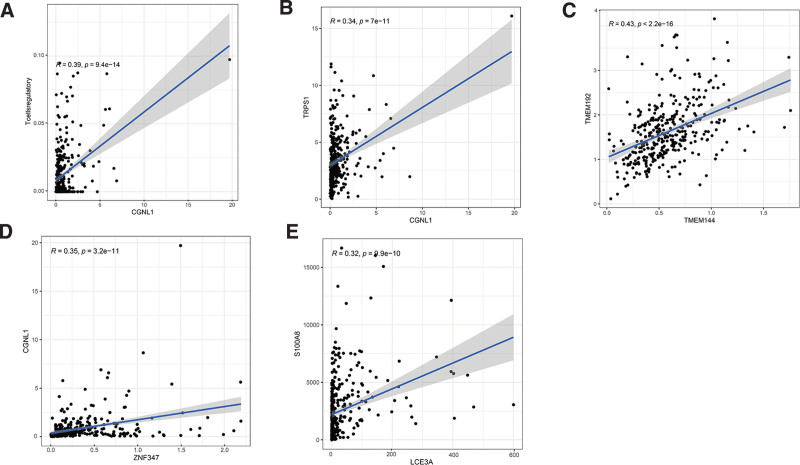
(A) Correlation between T cells regulatory and CGNL1 (*R* = 0.39, *P* < .001). (B) Correlation between TRPS1 and CGNL1 (*R* = 0.34, *P* < .001). (C) Correlation between TMEM144 and TMEM192 (*R* = 0.43, *P* < .001). (D) Correlation between CGNL1 and ZNF347 (*R* = 0.35, *P* < .001). (E) Correlation between LCE3A and S100A8 (*R* = 0.32, *P* < .001).

The Pearson’s correlation coefficient indicated the correlation between key RNAs and 22 types of lymphocytes in oral cancer samples (Fig. 8). It shows some essential co-expression patterns for key members of the ceRNA network and immune cells, including T cell regulatory and CGNL1 (*R* = 0.39, *P* < .001), TRPS1 and CGNL1 (*R* = 0.34, *P* < .001), TMEM144 and TMEM192 (*R* = 0.43, *P* < .001), CGNL1 and ZNF347 (*R* = 0.35, *P* < .001), LCE3A and S100A8 (*R* = 0.32, *P* < .001) (Fig. 9).

### 3.6. Multidimensional validation

We used a tumor database to analyze the expression levels of key genes in different types of cancer (*P* value < .01, |LogFC|>1.5, generank selected the top 10%). GGCT and HENMT1 are upwardly regulated in most cancers and lowly-regulated in a few cancers (Supplemental Digital Content [Fig. S6, http://links.lww.com/MD/I101]). CGNL1 and S100A8 are down-regulated in most cancers and up-regulated in a few cancers. TMEM144 and TRPS1 behave differently in different cancers. The data of TMEM192 and LCE3A in the oncomine database are too few to be compared and analyzed. In the TIMER2.0 database, compared with normal tissues, HENMT1 in head and neck cancer tissues was significantly up-regulated (*P* < .05), and ZNF347, S100A8, TMEM144, TRPS1, and CGNL1 were significantly down-regulated (*P* < .05, Supplemental Digital Content [Fig. S7, http://links.lww.com/MD/I102]). Survival analysis based on the Kaplan–Meier plotter website was used to assess their effect on the OS rate of oral cancer. The high expression rates of HENMT1, CGNL1, S100A8, TRPS1, LCE3A were all related to the better prognosis of patients (*P* < .05), and the high expressions of TMEM144 and TMEM192 are related to the poor prognosis of patients (*P* < .05) (Supplemental Digital Content [Fig. S8, http://links.lww.com/MD/I103]).

## 4. Discussion

The study of oral cancer biomarkers will help to provide a theoretical basis for the development of targeted drugs. Various genetic and molecular biomarkers, such as protein coding genes and non-coding genes, have been used in the diagnosis and treatment of oral cancer. Among them, ceRNA and tumor infiltrating immune cells have attracted people’s attention. The ceRNA network, including mRNA, miRNA, and lncRNA, and infiltrating immune cells may be critical to further development of targeted drugs. However, the underlying molecular mechanism between them is still unknown.

In this study, we discovered the differentially expressed ceRNA network between oral cancer and normal tissues, and identified key immune cells in the tumor micro-environment. A prognostic risk model was constructed based on the key genes and immune cells, and a nomogram was graphed. GGCT, TRPS1, CGNL1, HENMT1, LCE3A, S100A8, ZNF347, TMEM144, TMEM192, T cells regulatory and Eosinophils in the model can effectively predict prognosis. GGCT is the coding gene of gamma-glutamylcyclotransferase. Gamma-glutamylcyclotransferase is expressed higher than normal in many tumor tissues. The lack of GGCT in a variety of cancer cells can produce anti-proliferative effects, so it is considered a promising therapeutic target. Previous studies have proved that phasing out GGCT can induce cell senescence and non-apoptotic cell death. This effect is attributed to the upward regulation of cyclin-dependent kinase inhibitors including p21WAF1/CIP1. Cells lacking GGCT can trigger a signal cascading that promotes autophagy, including activation of the AMPK-ULK1 pathway and/or inactivation of the mTORC2-Akt pathway.^[23]^ Zinc finger transcription factor Trps1 is referred to as TRPS1. The expression of TRPS1 is regulated by hormones, and androgens can reduce the expression of TRPS1. High expression rates of TRPS1 can be observed in a variety of cancer tissues. It has been studied in prostate and breast cancer.^[24]^ CGNL1 is an endothelial junction complex protein that regulates GTPase-mediated angiogenesis.^[25]^ Besides, HENMT1 is a coding gene for Small RNA 2’-O-methyltransferase. Studies have shown that HENMT1 is poorly regulated in a variety of cancers.^[26]^ LCE3A is the coding gene of late cornified envelope protein 3A. Late cornified envelope protein 3A is a structural component of the keratinized membrane of the stratum corneum. It can regulate skin barrier repair through an immune response to bacterial antigens. S100A8 is the coding gene of Protein S100-A8. Studies have shown that inhibiting the expression of S100A8 and S100A9 in cancer cells can significantly reduce the migration and invasion of tumor cells during culture.^[27]^ ZNF347 is the encoded gene of Zinc finger protein 347.^[28]^ TMEM144 is a gene encoding Transmembrane protein 144.^[29]^ TMEM192 is a gene that encodes Transmembrane protein 192.^[30]^ However, there has been little research on the correlation between CGNL1, LCE3A, ZNF347, TMEM144, TMEM192, and cancer. They may be potential therapeutic targets and need to be identified in future studies.

The adaptive immune system is regulated by an important subset of CD4 + T lymphocytes, called regulatory T cells, to maintain immune homeostasis by preventing over-immune activation. Lack of Treg and excessive activation can lead to pathological changes in the disease. Although the loss of Treg function can lead to autoimmune activity, too much Treg activation can promote the occurrence of tumors. Blocking and/or removing Tregs has become a viable strategy to enhance anti-tumor immunity. New methods of improving the targeting specificity of Tregs in tumors include the use of T cell receptor mimic antibodies, bispecific antibodies, and near-infrared immunotherapy. Currently, a number of screening methods based on transcriptomics have been developed to determine the preferred Treg targets.^[31]^ Eosinophils are a kind of multi-functional immune cells involved in the pathogenesis of many inflammatory processes. In many human cancers, including oral squamous cell carcinoma, its presence is related to the prognosis of the patient. Although the exact role of eosinophils in tumors is not clear, the anti-tumor activity of these rare granulocytes is associated with the release of cytotoxic proteins, especially eosinophil cationic protein. Functional studies have demonstrated that eosinophil cationic protein is participated in many processes, such as tissue remodeling of allergic inflammation, but its most significant function is cytotoxicity. This study and results show that a high level of eosinophils is associated with a poor prognosis, and a high level of T cells regulatory is associated with a better prognosis.^[32]^ As it turns out, it is shown that high concentrations of eosinophils are associated with poor prognosis, while high levels of T cells regulatory are associated with better prognosis.

In the co-expression relationship, T cells regulatory and CGNL1 illustrated a significant positive correlation. Our results show that patients with high levels of T cells regulatory have a better prognosis. CGNL1 in tumor tissues was significantly lower than that in adjacent tissues, and patients with high levels of CGNL1 have a better prognosis. The final correlation analysis results are consistent with these studies. T cells regulatory and CGNL1 (*R* = 0.39, *P* < .001) showed a significant positive correlation. We speculate that this pair of co-expression mechanism may play an important role in the treatment of oral cancer. However, after a systematic literature review, no clear report was found on the correlation between T cells regulatory and CGNL1. The exact correlation between T cells regulatory and CGNL1 in the development of oral cancer has yet to be confirmed in future studies. Our research helped to identify these relationships and laid the foundation for their in-depth research.

There are inevitably some limitations in our study that must be acknowledged. First of all, limited number of samples we collect from public databases means that the clinical pathological parameters analyzed in our research are incomplete and may bring some locality.^[33]^ In addition, we did not consider that the heterogeneity of the immune microenvironment is related to the location of immune infiltration.^[34]^ Last but not least, the biggest problem in this study lies in the lack of verification of key genetic mechanisms. However, in order to reduce this bias, we also used multiple databases to reveal the gene expression of key biomarkers in tumors and normal tissues.

## 5. Conclusion

Basing on the ceRNA network and tumor-infiltrating immune cells we studied, we constructed 2 multivariate prognostic risk models to predict the prognosis of oral cancer patients. The higher AUC value proves the accuracy of our model. Moreover, our study inferred that patients with high levels of T cells regulatory and CGNL1 have a better prognosis and lower risk. Co-expression analysis also indicated that T cells regulatory and CGNL1 have a significant positive correlation. This result suggests that T cells regulatory and CGNL1 may play an important role in the prognosis of oral cancer.

## Acknowledgments

The authors gratefully acknowledge the support of this work by Key science and technology research projects of Health and Family Planning Commission of Hebei Province (NO. 20210801; NO. 20170737).

## Authors contributions

**Conceptualization:** Tianke Li.

**Data curation:** Sai Ma, Jie Guo, Xuan Zhang, Yongchao Yang, Tianke Li.

**Formal analysis:** Sai Ma, Jie Guo, Yang Bao.

**Funding acquisition:** Tianke Li.

**Investigation:** Sai Ma, Xuan Zhang, Tianke Li.

**Methodology:** Sai Ma, Jie Guo, Yongchao Yang.

**Project administration:** Jie Guo, Tianke Li.

**Resources:** Tianke Li.

**Software:** Sai Ma, Suxin Zhang.

**Supervision:** Sai Ma, Suxin Zhang.

**Validation:** Sai Ma, Jie Guo.

**Visualization:** Sai Ma, Xuan Zhang, Yang Bao.

**Writing – original draft:** Sai Ma.

**Writing – review & editing:** Tianke Li.

## Supplementary Material


