# Supplementary material for: The exploration of new biomarkers for oral cancer through the ceRNA network and immune microenvironment analysis
Source: Medicine (Baltimore). 2022 Dec 9;101(49):e32249. doi: 10.1097/MD.0000000000032249 (PMC9750585; doi:10.1097/MD.0000000000032249)
Supplement: Supplementary file 1 [file medi-101-e32249-s001.pdf]

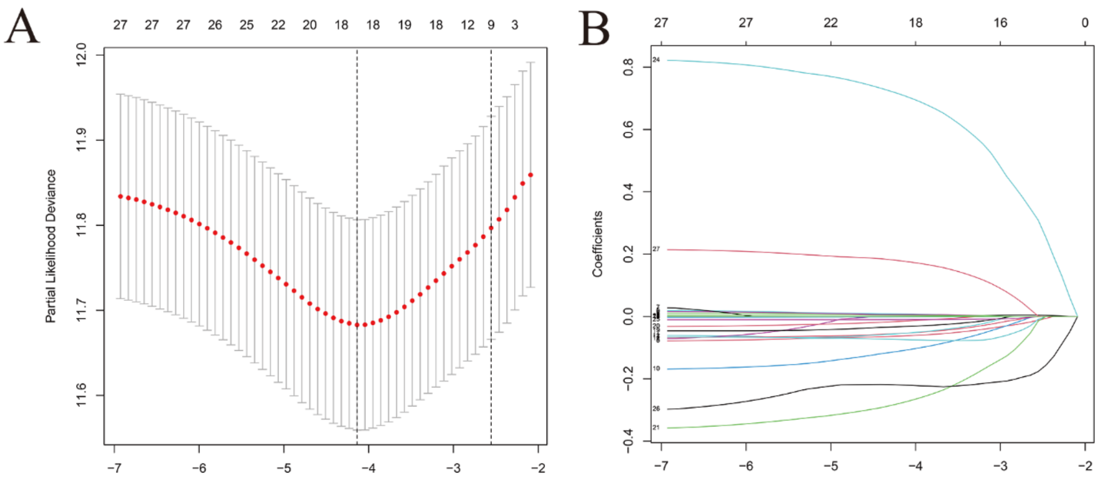

Figure S1 (A)The confidence interval under each lambda. (B) The change trajectory of each independent variable. The horizontal axis represents the log value of the independent variable lambda, and the vertical axis represents the coefficient of the independent variable.
