# Supplementary material for: The exploration of new biomarkers for oral cancer through the ceRNA network and immune microenvironment analysis
Source: Medicine (Baltimore). 2022 Dec 9;101(49):e32249. doi: 10.1097/MD.0000000000032249 (PMC9750585; doi:10.1097/MD.0000000000032249)
Supplement: Supplementary file 3 [file medi-101-e32249-s003.pdf]

A

B

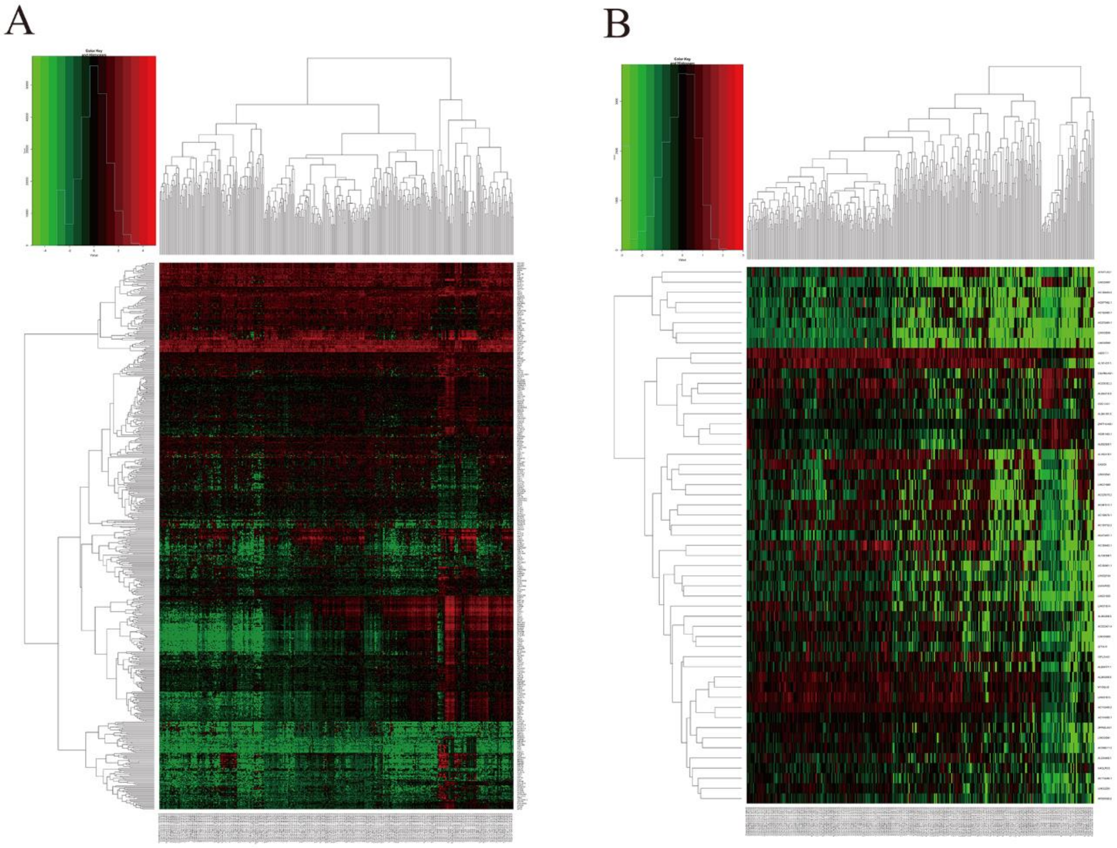

Figure S3 (A) The heatmap of differentially expressed mRNAs between normal and oral cancer tissues. (B)The heatmap of differentially expressed lncRNAs between normal and oral cancer tissues.
