# Supplementary material for: The exploration of new biomarkers for oral cancer through the ceRNA network and immune microenvironment analysis
Source: Medicine (Baltimore). 2022 Dec 9;101(49):e32249. doi: 10.1097/MD.0000000000032249 (PMC9750585; doi:10.1097/MD.0000000000032249)
Supplement: Supplementary file 4 [file medi-101-e32249-s004.pdf]

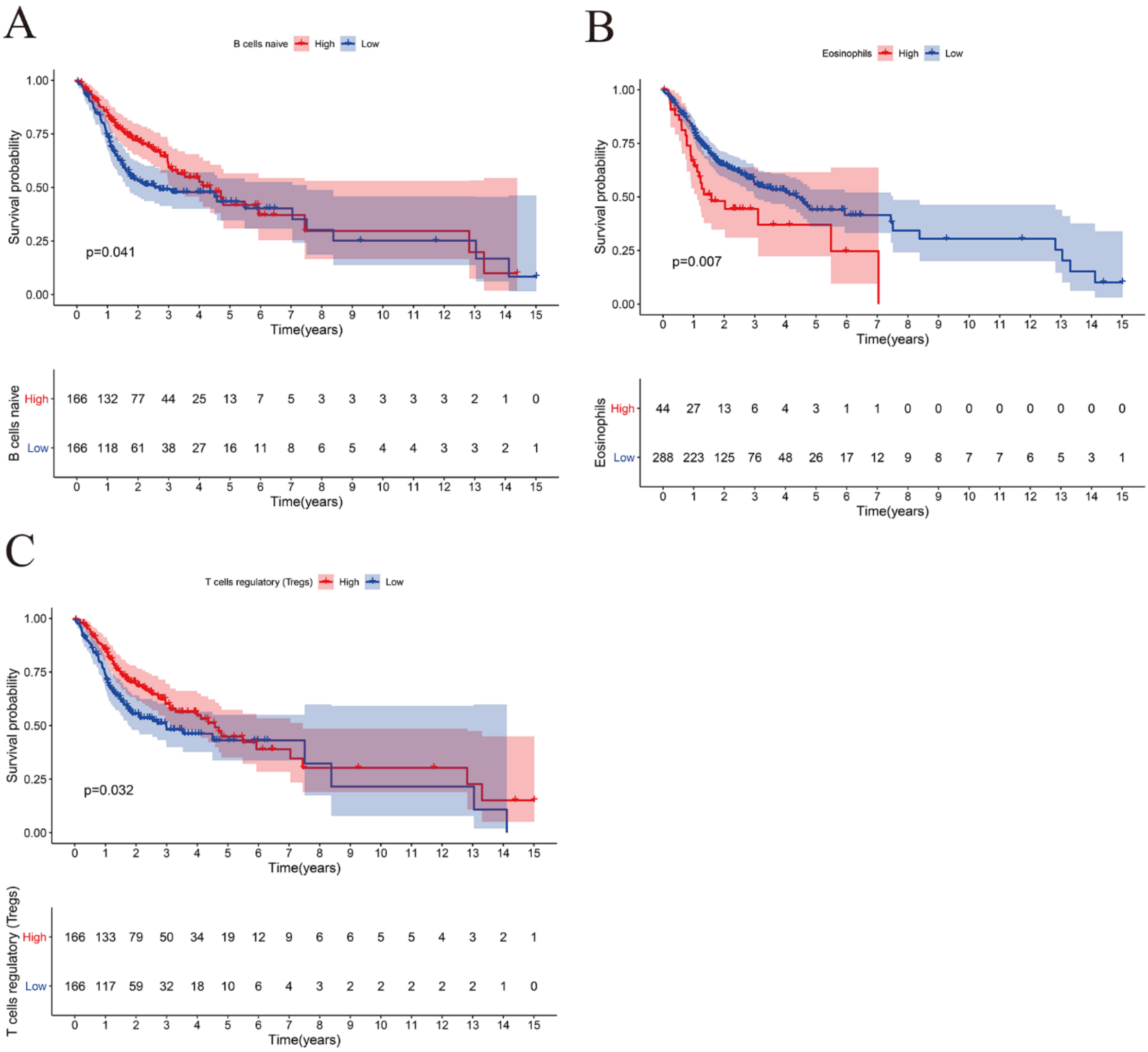

Figure S4 (A)The relationship between B cells naive and prognosis of oral cancer patients. (B)The relationship between Eosinophils and prognosis of oral cancer patients. (C)The relationship between T cells regulatory and prognosis of oral cancer patients.
