# Supplementary figures and images for: The exploration of new biomarkers for oral cancer through the ceRNA network and immune microenvironment analysis
Source: Medicine (Baltimore). 2022 Dec 9;101(49):e32249. doi: 10.1097/MD.0000000000032249 (PMC9750585; doi:10.1097/MD.0000000000032249)

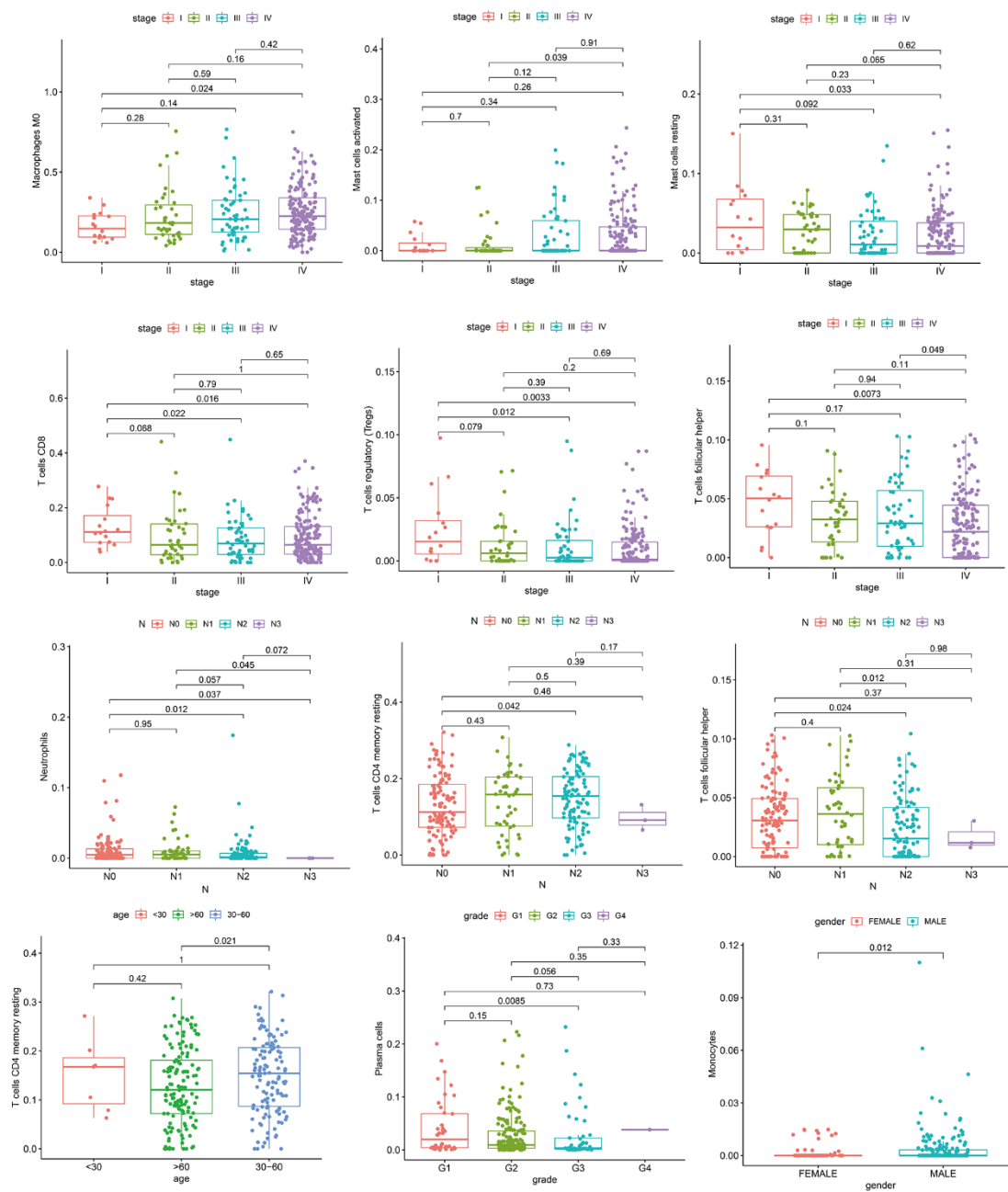

Figure S5 Box plots of the relationship between clinical features and immune cells.

Supplement: Supplementary file 5 [file medi-101-e32249-s005.pdf]

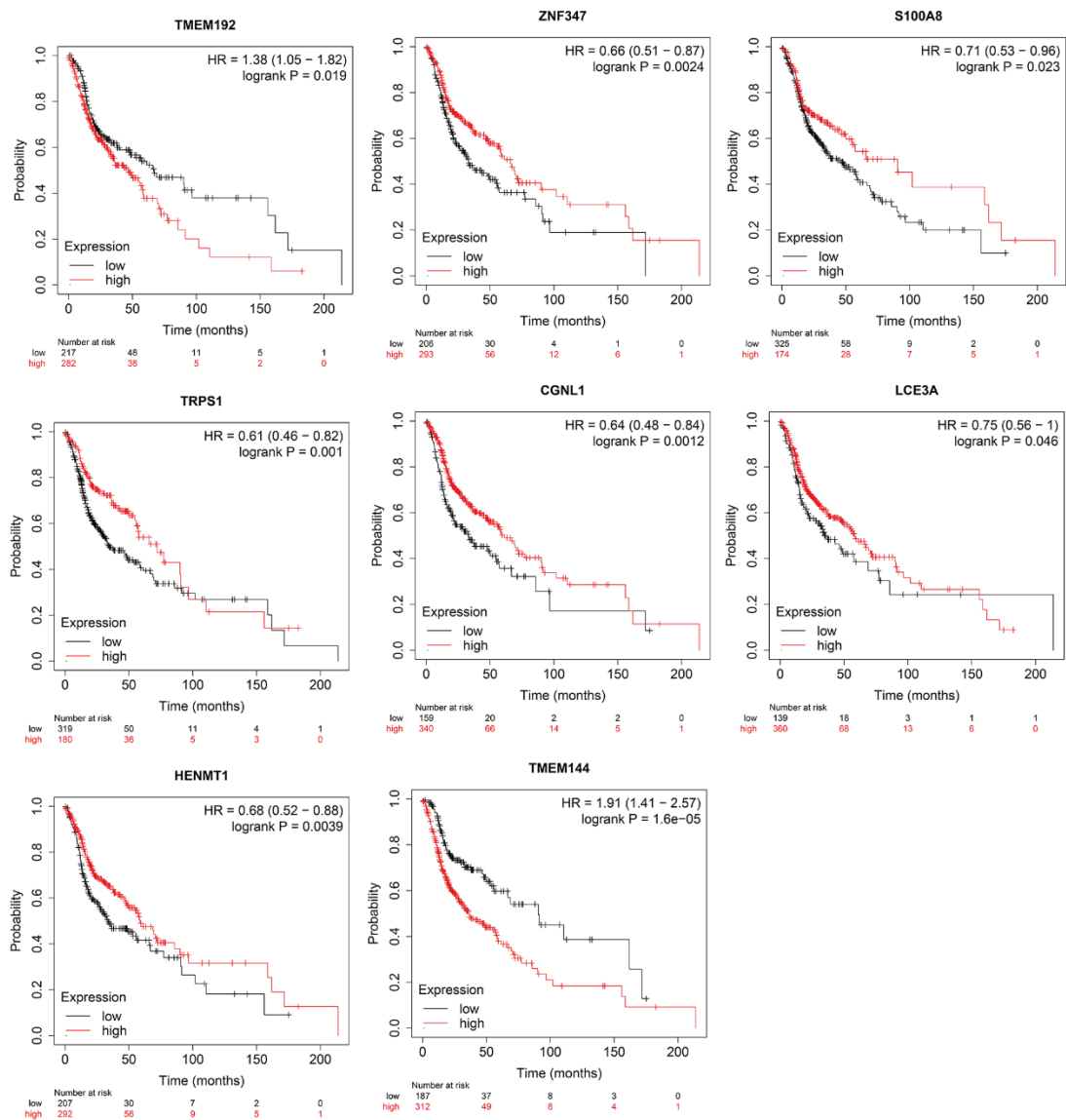

Figure S8 Survival analysis of key genes in Kaplan Meier plotter website.

Supplement: Supplementary file 8 [file medi-101-e32249-s008.pdf]
