# Supplementary material for: The exploration of new biomarkers for oral cancer through the ceRNA network and immune microenvironment analysis
Source: Medicine (Baltimore). 2022 Dec 9;101(49):e32249. doi: 10.1097/MD.0000000000032249 (PMC9750585; doi:10.1097/MD.0000000000032249)
Supplement: Supplementary file 6 [file medi-101-e32249-s006.pdf]

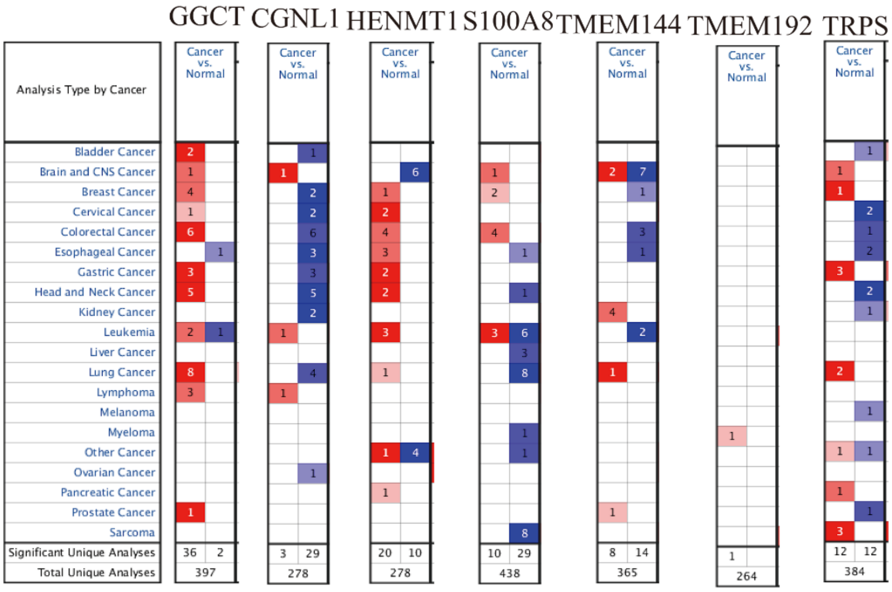

Figure S6 The differential expression of key genes in different tumors was analyzed in oncomine database. The color in the box represents up (red) or down (blue) regulation in the tumor.
