# Supplementary material for: The exploration of new biomarkers for oral cancer through the ceRNA network and immune microenvironment analysis
Source: Medicine (Baltimore). 2022 Dec 9;101(49):e32249. doi: 10.1097/MD.0000000000032249 (PMC9750585; doi:10.1097/MD.0000000000032249)
Supplement: Supplementary file 7 [file medi-101-e32249-s007.pdf]

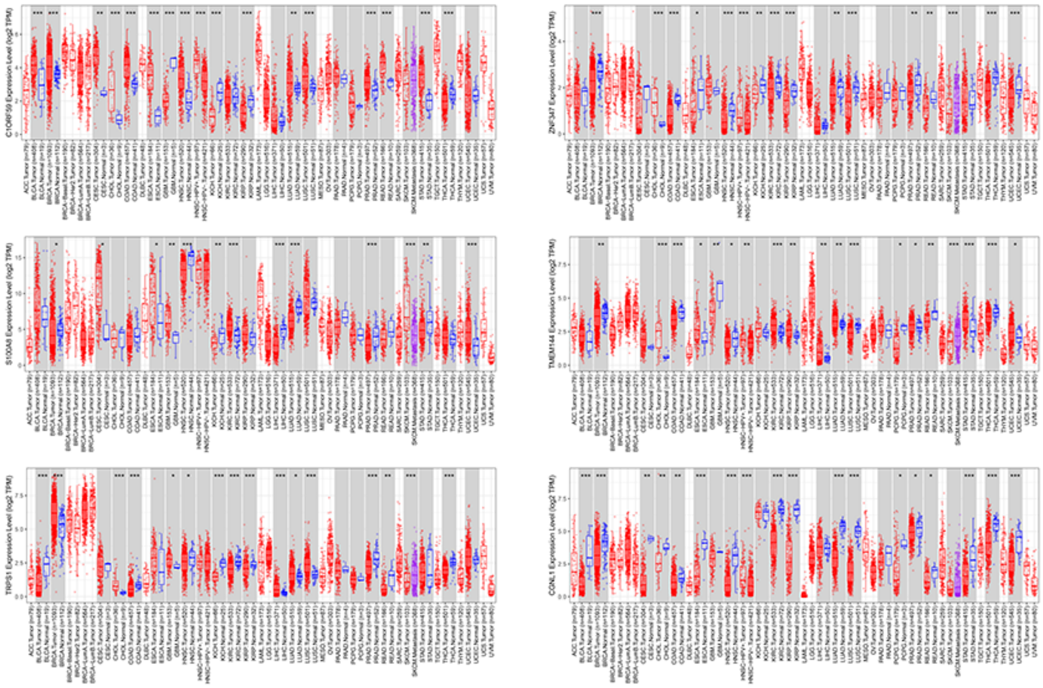

Figure S7. The differential expression of key genes in different tumors was analyzed in TIMER 2.0 database.
